# Evaluating the Impact of Telehealth Exercise Prehabilitation on Cardiometabolic Health in Bariatric Surgery Candidates: Protocol for the BARI-Prehab Randomized Controlled Trial

**DOI:** 10.2196/77538

**Published:** 2025-11-13

**Authors:** Belinda Jayne Durey, Alison M Coates, Kade Davison, Brett Tarca, Jessica Mok, Chetan D Parmar, Naiara Fernandez-Munoz, Katarina Burton, Nicholas Tetlow, Amy Louise Dewar, Mariam Olaide Adeleke, Zoe Lugg, Jack Colbert, Daniel S Martin

**Affiliations:** 1 Allied Health and Human Performance Alliance for Research in Exercise, Nutrition and Activity University of South Australia Adelaide Australia; 2 Faculty of Medicine and Health UNSW Sydney Sydney Australia; 3 Division of Surgery and Interventional Science University College London London United Kingdom; 4 Homerton University Hospital NHS Foundation Trust London United Kingdom; 5 Department of Surgery Whittington Health NHS Trust London United Kingdom; 6 Department of Anaesthesia and Peri-operative Medicine University College London Hospitals NHS Foundation Trust London United Kingdom; 7 Department of Statistical Science University College London London United Kingdom; 8 Peninsula Medical School University of Plymouth Plymouth United Kingdom; 9 Intensive Care Unit University Hospitals Plymouth Plymouth United Kingdom

**Keywords:** bariatric surgery, cardiorespiratory fitness, digital healthcare, exercise, obesity, perioperative care, prehabilitation, telehealth

## Abstract

**Background:**

Obesity affects over one billion people globally and is a leading contributor to chronic disease. For those with clinically severe obesity, metabolic and bariatric surgery (MBS) is the most effective intervention for long-term weight loss. However, surgery is often delayed due to systemic barriers, during which time patients may experience further health decline. Low cardiorespiratory fitness is a known risk factor for perioperative complications, prompting recommendations for prehabilitation to target readiness for surgery. Despite this, few patients meet physical activity guidelines, and supervised preoperative exercise programs are rarely offered in routine care. Telehealth-delivered exercise programs offer a promising solution, but evidence of their feasibility, acceptability, and impact in the MBS setting remains limited.

**Objective:**

This study (BARI-Prehab) aims to assess the efficacy and acceptability of a telehealth-delivered prehabilitation exercise program in improving cardiometabolic health among patients awaiting MBS.

**Methods:**

In this multicenter, open-label, randomized controlled trial, we will randomize 48 adult participants (1:1) to either usual care (control group) or a 4-week telehealth exercise intervention. The primary outcome is aerobic capacity (VO_2_ in mL/kg/min at the anaerobic threshold), measured using cardiopulmonary exercise testing. Secondary outcomes include resting heart rate, heart rate variability, resting metabolic rate, body composition, grip strength, and 7-day physical activity. Intervention acceptability will also be evaluated.

**Results:**

Data collection and analysis are ongoing. This trial, funded in September 2020, will evaluate the capacity of a telehealth exercise program to improve cardiometabolic health and determine its suitability for implementation in the MBS preoperative pathway. Following initial protocol development, ethics approval, and trial setup, the clinical phase formally commenced with registration on June 16, 2023. Enrollment is ongoing, with a projected end date of March 2026. As of May 2025, a total of 220 patients have been screened for participation, of whom 30 have enrolled in the trial. The first results are expected to be submitted for publication by mid-2026.

**Conclusions:**

The BARI-Prehab trial will provide evidence on the acceptability and impact of a remotely delivered exercise intervention in the context of MBS. These findings will have implications for the design of accessible, scalable preoperative care models. The significance of this research lies in its potential to guide clinical practice, inform policy, and improve health outcomes for patients undergoing MBS.

**Trial Registration:**

ClinicalTrials.gov NCT05235945; https://clinicaltrials.gov/study/NCT05235945

**International Registered Report Identifier (IRRID):**

DERR1-10.2196/77538

## Introduction

### Background

In 2022, over one billion people were classified as living with obesity, making it a well-recognized and critical global public health concern [1]. A multitude of individual [2], community [3], and countrywide [4,5] weight-management initiatives have been used; yet, the epidemic continues. For those living with clinically severe obesity (BMI ≥35 kg/m^2^, or ≥30 kg/m^2^ with comorbidities), metabolic bariatric surgery (MBS) has been shown to be the most effective treatment for weight loss compared to nonsurgical interventions [6,7]. However, eligible patients often face barriers to accessing surgery, such as extended wait-list times and costs [8]. Since obesity is a chronic progressive disease, it is likely that, without intervention, body mass and associated comorbidities may be negatively impacted by delays to surgery.

Surgical procedures, regardless of type, constitute a traumatic disruption to the body and are associated with a measurable stress response, which varies in intensity depending on the severity of the trauma [9]. Cardiorespiratory fitness (CRF) is considered a key indicator to predict the ability of a patient to withstand surgical injury in an adaptive manner [10]. For patients undergoing MBS, a lower CRF (<15.8 mL/kg/min) is associated with an increased risk of perioperative complications, including myocardial infarction, deep vein thrombosis, pulmonary embolism, renal failure, or death [11]. Current guidelines for Enhanced Recovery After Surgery recommend tailored preoperative physical activity (PA) interventions, or “prehabilitation,” in the lead-up to MBS to assist with patients’ functional recovery [12]. Improving PA levels has been reported to play an important role in postsurgical weight loss and its maintenance [13], in addition to well-established long-term physical and mental health benefits for people with obesity [14,15]. The timing of exercise programs before surgery is also important for establishing healthy behaviors, maintaining lean tissue, and mitigating potential decreases in metabolic rate [16]. Moreover, during this period, patients are yet to face challenges of recovering from surgery, including postoperative diet adaptation and loss of muscle strength.

The majority of MBS candidates do not meet PA recommendations, and consequently, often have associated reduced levels of CRF [17,18]. It has been established that exercise interventions in individuals with obesity should involve a combination of vigorous-intensity aerobic and high-load resistance training for the improvement of CRF [19]. In clinical populations, alternating short periods of vigorous-high intensity exercise with rest or low-intensity periods has been shown to be safe and effective for improving cardiometabolic risk factors and CRF in as little as 2 weeks [20-22].

Despite this, there is limited evidence supporting exercise services as a standard treatment in perioperative care for MBS patients. Supervised exercise programs are not commonly practiced in Australia or the United Kingdom, and unsupervised programs often have low participation rates [23,24]. Previous studies have identified several barriers reported by both clinicians and patients, including cost, limited access to exercise facilities, and time constraints [25]. Telehealth has emerged as a practical solution to address such barriers [26,27]. Evidence from recent studies supports the feasibility and acceptability of telehealth in MBS candidates [28-32]. However, these have typically focused on postsurgical populations, used group-based formats, or relied primarily on unsupervised digital resources. While these studies show promise for telehealth approaches, they also reveal a gap in evidence regarding the impact of supervised, individualized, preoperative exercise interventions for MBS candidates.

Several trials have been conducted to assess the effectiveness of a variety of exercise programs for patients with obesity, both before and after MBS. Overall, the evidence is limited by small sample sizes, insufficient long-term follow-up (<1 year), and differing methodologies and time frames for assessment [33,34]. Body mass change is often the primary outcome, while objective measures of fitness and PA, such as cardiopulmonary exercise testing (CPET) and accelerometry, have rarely been reported [33]. While the evidence in MBS is sparse, preoperative exercise and improved fitness in patients with obesity have been associated with reductions in cardiometabolic risk and early death [11]. Given the rising prevalence and detrimental impact of obesity, there is a need to explore strategies that can mitigate unfavorable outcomes in the perioperative period. Exercise prehabilitation may offer a scalable and impactful approach to improving patient readiness and resilience. Integration of such models into routine care pathways remains underexplored. This study therefore aims to evaluate the effect of a structured, telehealth-delivered prehabilitation exercise intervention on cardiopulmonary fitness in patients awaiting MBS.

### Objectives

This randomized controlled trial, BARI-Prehab, aims to evaluate the efficacy of an exercise intervention in the preoperative MBS setting. Specifically, we will (1) objectively measure changes in cardiometabolic health–related parameters with an exercise intervention and (2) determine the acceptability of an online–delivered exercise program for patients preparing for MBS before surgery.

## Methods

### Ethical Considerations

Ethics approval has been granted by the Derby Research Ethics Committee (21/EM/0230) and the University of South Australia Human Research Ethics Committee (204474). The trial was registered on January 12, 2022, in ClinicalTrials.gov (NCT05235945). Each potential participant is given at least 24 hours to decide whether to participate after receiving the information sheet. Those who decide to participate will be invited to the laboratory to sign the consent form on the morning of their scheduled preassessment anesthetic clinic visit. They will receive a hard copy of both the information sheet and signed consent form and will have the opportunity to ask any study-related questions. Participants are free to withdraw at any time without providing a reason and without this affecting any benefits to which they are entitled. To ensure confidentiality, all identifiable data will be stored in a password-protected document or in a locked filing cabinet. The chief investigator is the custodian of personal data. Access to storage and premises will be limited to research team members only. All data will be anonymized and pseudonymized. A unique ID will be assigned to each participant once enrolled in the study. Access to participants’ personal data will be restricted to research personnel only. Only anonymous data will be used in publications or presentations. The University College London (UCL) Data Protection Policy will be adhered to. Participant engagement strategies will be implemented, including compensation for travel expenses and provision of an exercise band (used during the intervention) to support adherence.

### Trial Design

**Figure 1:**
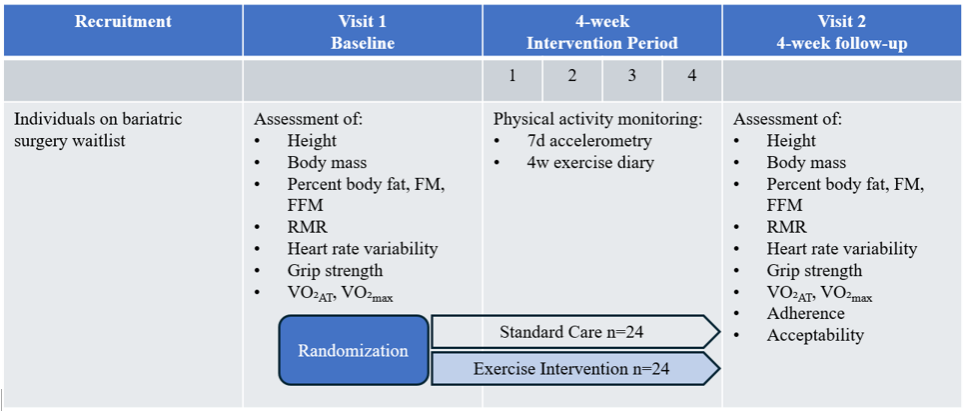
BARI-Prehab study design. Abbreviated assessment items include fat mass (FM), fat-free mass (FFM), resting metabolic rate (RMR), volume of oxygen consumption at anaerobic threshold (VO_2__AT_), and maximal oxygen consumption (VO_2__max)_. Physical activity is monitored throughout the intervention period using 7-day (7d) accelerometry, and 4-week (4w) recall using an exercise diary.

BARI-Prehab is a multicenter, open-label, randomized controlled trial designed to test the effect and acceptability of a novel preoperative exercise program in patients scheduled for MBS. The sponsor for this trial is University College London Hospitals (UCLH) National Health Service (NHS) Foundation Trust. Participants will be randomized to either a control group (usual care) or a 4-week supervised exercise intervention group, using a 1:1 allocation ratio. The study flow diagram is shown in Figure 1, and Table 1 outlines the exercise intervention. The Standard Protocol Items: Recommendations for Interventional Trials (SPIRIT) reporting guidelines [35] have been used.

**Table 1:** BARI-Prehab trial intervention session design. Exercises are tailored to match each participant’s abilities, with the goal of reaching a perceived exertion (RPE) level of 5/10 (described as “hard”). Intensity is achieved by modifying factors such as range of motion (ROM), additional muscle groups engaged, or movement speed.

Phase	Exercise	Level 1	Level 2	Level 3
Warm-up (5 minutes)	Compound movements involving large muscle groups and dynamic stretches	—^a^	—	—
1	Sit-to-stand	Supported (wall)	Unsupported	Compound (+arms)
2	Wall push-up	Isometric	Full ROM	Plyometric
3	Lunge twist	Tap back	Supported (chair/wall)	Compound (+arms)
4	Standing oblique crunch	Supported (chair)	Unsupported	Increased ROM/speed
5	Deadlift + bent-over row	Without band	With band	Increased ROM
6	Walking squat	Isometric	Increased ROM	Compound (+arms)
7	Side reaches	Supported (chair)	Unsupported	Increased ROM/speed
8	Shoulder flexion + balance	Without band	With band	Increased ROM/speed
9	Arabesque/Standing hamstring curl	Supported (chair/wall)	—	Unsupported
10	Forward jabs (boxing)	Without band	With band	Compound (+legs)
Cool-down (5 minutes)	Dynamic and static stretches	—	—	—

^a^Not applicable.

### Eligibility Criteria

Participants will consist of adults aged ≥18 years who are enrolled in the bariatric surgery programs at selected sites, with BMI ≥30 kg/m^2^. Patients must have ≤5% variation in body weight over the preceding 3 months, be willing and able to comply with the trial protocol, and provide written informed consent.

A patient who meets any of the following criteria will be excluded from participation in this study: currently pregnant or lactating, body mass ≥180 kg (due to weight restrictions of equipment), currently using beta-blockers, participating in another clinical intervention trial, clinically significant medical comorbidities present (eg, uncontrolled hypertension, unstable cardiovascular disease) that could increase the risk of an adverse response to exercise [36], or a history of atrial fibrillation, unstable angina, acute coronary syndrome, or congestive heart failure (New York Heart Association class III–IV) within the preceding 12 months.

### Sample Size

We will aim to enroll 48 participants. The sample size for this study was determined by detecting a clinically meaningful increase in anaerobic threshold (AT) in patients awaiting MBS. Previous research in a comparable patient cohort [37] reported a mean AT of 11.0 (SD 1.5) mL/kg/min among 95 patients. In this study, we aim to detect a 150 mL O_2_ increase in AT, corresponding to a mean difference of 1.15 mL/kg/min between the two groups. This threshold was selected based on literature indicating that changes of approximately 0.75-1.25 SDs in AT are associated with improved perioperative risk profiles and functional capacity in surgical populations [37-39]. We calculated that a sample size of 21 participants per group would be sufficient to detect a 150 mL O_2_ increase in AT, with a significance level of .05 and a power of 0.80. This calculation is based on having 2 repeated measurements per participant and an intracluster correlation of 0.5. Pilot research by Gilbertson et al [40] has informed our anticipated dropout rate of less than 10%, or 2 participants per treatment arm.

### Recruitment

Eligible participants will be identified by the clinical team from the operating lists of recruiting hospitals, which are established at least 6 weeks before surgery. Identified patients will then be invited for enrollment by phone, where they are contacted by a research team member and provided with a participant information sheet. Patients will be offered opportunities to ask questions about the study and given time to consider enrolling. Recruitment will occur on a rolling basis at a rate of 5 patients per month. Recruitment rate assumptions will account for anticipated challenges, including those specific to the bariatric population. These may include mobility limitations, comorbid conditions, or logistical issues associated with travel for study assessments [25].

### Allocation

Following enrollment, participants will attend a baseline testing visit (visit 1), where they will be allocated a unique subject number. Once baseline data collection has taken place, participants will be randomly assigned using an electronic third-party platform [41] to a treatment group—preoperative exercise intervention or usual care—on a 1:1 allocation basis. A member of the research team will generate the concealed allocation sequence, while a second researcher will assign participants to interventions. Members of the research team responsible for conducting preassessments and postassessments will be blinded to allocation. Participants and clinicians will not be blinded to allocation during the study, although all data will be analyzed blind to allocation.

### Interventions (Preoperative Exercise Group Only)

A 4-week structured exercise program will be delivered online using a cloud-based video conferencing service [42]. Participants in the exercise intervention group will individually undertake 7 instructor-led exercise sessions across the 4-week intervention period. In addition, participants will be provided with existing digital guidelines for presurgery exercise [43] and encouraged to participate in self-led PA on as many days as possible during the 4-week period. To guide this, participants will have access to prerecorded online exercises and are encouraged to replicate supervised sessions during each self-led session. The program is designed to meet the National Physical Activity Guidelines [44] of at least 150 minutes of low-to moderate-intensity or 75 minutes of vigorous PA per week, or a combination of the two [45]. Participants will be asked to record daily PA in an electronic diary, alongside a standardized exercise-related rating of perceived exertion [46] for each session and duration of exercise participation.

Sessions will be supervised by an accredited exercise physiologist, who is a university-qualified allied health professional with training in the design and delivery of safe and effective exercise interventions for people with chronic medical conditions, injuries, or disabilities [47]. Participants are offered a range of scheduling options to accommodate varying availability, including morning and afternoon time slots on both weekdays and weekends, with options within and outside standard business hours. If an appointment is canceled or missed, our protocol permits rescheduling within a 5-day window to maintain consistency with study timelines.

Each supervised session (Table 1) will consist of aerobic and resistance exercises, targeting all main muscle groups and tailored to participants based on their exercise history and limitations. Participants’ baseline functional level will guide the targeted exercise prescription. Sessions will be prescribed at moderate- to vigorous-intensity (Table 2) for a 30-minute duration. A set of varying-resistance exercise bands will be provided for participants.

Intervention-group participants will receive weekly check-ins by text, emails, or phone call. The check-ins are designed to promote adherence to both the intervention program and their PA sessions. A summary of the content of participant communications is presented in Table S1 in Multimedia Appendix 1 [48-50], and is based on behavior change techniques such as goal setting, individualized feedback, and social incentive (ie, positive reinforcement), which have been shown to improve exercise participation in digital interventions [48-50].

Participants in the comparator group will receive the usual care provided at their respective treatment sites. This care varies across sites. It ranges from no formal intervention from the clinical team to inclusion of nutritional and psychological support but does not routinely involve structured or supervised exercise.

**Table 2:** Categories of exercise intensity and associated objective and subjective measures, such as percent of maximum heart rate (%HRmax), percent heart rate reserve (%HRR; heart rate reserve=HRmax−resting heart rate), and percent of maximal oxygen uptake (%VO_2_max). Note: the relative intensity measures will not always correspond to the same rating of perceived exertion (RPE) among individuals, nor will the ability of participants to exercise for a specific duration at each intensity, since this varies depending on training status and other personal characteristics. Subjective measures are from Borg’s [46] RPE scales, where C=category scale (6-20) and C-R=category-ratio scale (0-10). Adapted with permission from Norton et al [45].

Intensity	Objective measures	Subjective measures	Descriptive measures
Sedentary	<1.6 METsa <40%HRmax <20% HRR <20% V02max	RPE (6-20): <8 RPE (0-10): <1	Low energy requirement, such as sitting or lying activities
Light	1.6<3 METs 40<55% HRmax 20<40% HRR 20<40% V02max	RPE (6-20): 8-10 RPE (0-10): 1-2	Aerobic activities that do not cause a noticeable change in breathing rate or can be sustained for ≥60 minutes
Moderate	3<6 METs 55<70% HRmax 40<60% HRR 40<60% V02max	RPE (6-20): 11-13 RPE (0-10): 3-4	Aerobic activities that feel “light” to “somewhat hard” and can be conducted while maintaining a conversation; duration 30-60 minutes
Vigorous	6<9 METs 70<90% HRmax 60<85% HRR 60<85% V02max	RPE (6-20): 14-16 RPE (0-10): 5-6	Aerobic activities during which a conversation cannot be maintained uninterrupted; duration up to 30 minutes
High	>9 METs >90% HRmax >85% HRR >85% V02max	RPE (6-20): >17 RPE (0-10): >7	Activities that feel “very hard” and generally cannot be sustained for longer than 10 minutes

^a^MET: metabolic equivalent of task.

### Outcomes

The primary outcome is oxygen consumption at the AT, expressed relative to body mass (mL/kg/min) or in absolute terms (mL/min), attained from maximal CPET. AT is a submaximal gas exchange threshold defined as “the VO_2_ above which anaerobic metabolism supplements oxidative phosphorylation with additional carbon dioxide (CO_2_) production” [51]. The gold standard method for determining AT is the V‐slope method [52], which identifies the first inflection point on the plot of CO_2_ output versus O_2_ uptake. Secondary outcomes, technical and data processing procedures, and equipment used are detailed in Table S2 in Multimedia Appendix 2.

At visit 1, all participants will undergo assessment of height, body weight and composition, resting heart rate and heart rate variability, resting metabolic rate, CRF, and grip strength. Participants will also be given an accelerometer to measure low-, moderate-, and vigorous-intensity PA patterns and sedentary behavior over a 7-day period. At the end of the 4-week intervention period, the baseline (visit 1) tests will be repeated for comparison (visit 2).

Following randomization, participants will receive an email containing allocation-specific instructions for the trial period and an electronic exercise diary. Participants in the intervention group will additionally receive a troubleshooting guide for online classes and a timetable of options for booking exercise sessions.

#### Acceptability

Intervention acceptability will be established by the Participant Satisfaction Survey responses on a 5-point Likert scale, where aspects of the intervention such as duration of sessions, equipment used, exercises included, supervision, and delivery mode are rated from “liked very much” to “disliked very much.” This is measured only in the intervention group. Open-ended responses are requested for questions such as “What, if anything, would you change about the pre-surgery exercise program?”

#### Data Management

In accordance with UCL Data Protection Policy, study data will be collected and managed using the Research Electronic Data Capture tool [53] hosted at UCL. All data forms will be deidentified and paper copies will be double-entered and stored in the username- and password-protected electronic database. Electronic personal data will be stored separately from the study data in a password-protected document on encrypted UCL and NHS desktop computers. Pseudo-anonymized data will be stored on password-protected and encrypted UCL and NHS desktop computers.

#### Statistical Analysis Plan

Descriptive statistics will be used to summarize baseline characteristics and the primary outcome by treatment group, using mean (SD) and median (IQR) for continuous data and frequency (%) for categorical data. The distribution of the primary outcome will also be graphically investigated.

Treatment effect will be presented as the estimate and a 95% CI. This will be estimated from a linear mixed regression model with repeated measures of the outcomes at baseline and 4 weeks, with a random intercept for participants to account for correlations between repeated measures over time within participants. The model will include, as fixed effect a treatment arm indicator (during the follow-up phase only) and a time indicator (baseline or follow-up). All analyses will be performed on an intention-to-treat basis, and all modelling assumptions will be checked (eg, residuals).

A complier average causal effect estimate will be calculated to investigate potential effect of non-compliance. The number (%) of participants with missing outcomes will be summarized by treatment arm. A sensitivity analysis that includes predictors of missingness as covariates in the primary analysis model will be conducted. Multiple imputation will also be performed as an additional sensitivity analysis for missing data. A per-protocol analysis will also be carried out as a sensitivity analysis. The effect of the intervention on secondary outcomes will be assessed using methods analogous to those used for the primary outcome.

Satisfaction survey responses from the intervention group will be summarized descriptively. Where appropriate, exploratory analyses will examine variation in responses based on participant characteristics (eg, age, baseline fitness, engagement in sessions) to identify factors associated with higher acceptability or satisfaction.

#### Monitoring

The chief investigator will be responsible for the day-to-day monitoring and management of the study. The UCLH/UCL Joint Research Office, on behalf of UCL as Sponsor, will monitor and conduct random audits on a selection of studies in its clinical research portfolio. Monitoring and auditing will be conducted in accordance with the Department of Health Research Governance Framework for Health & Social Care [54] and in accordance with the sponsor’s monitoring and audit policies and procedures.

#### Data Monitoring

In accordance with the UCL Records Retention Policy, at the end of the trial, all essential documentation will be archived securely by the chief investigator for a minimum of 20 years from the declaration of the end of trial. After 20 years, stored data will be securely destroyed.

#### Harms

In alignment with SPIRIT guidelines [35], adverse event monitoring and handling procedures have been established to ensure participant safety. Given the telehealth nature of the intervention, specific adaptations have been made to address challenges related to remote supervision, participant adherence, and emergency response. Participants are required to complete thorough pre-exercise screening, and real-time monitoring is conducted through online sessions by the supervising exercise physiologist. Additionally, clear protocols are in place for identifying and responding to adverse events, including escalation procedures for medical concerns and access to emergency services if needed. For research staff, there is a potential risk of sustaining an injury when assisting patients on and off the exercise equipment; however, this is part of the routine procedure in exercise testing, and only experienced staff will undertake this role. Manual handling and intermediate life support training have been completed by all involved staff.

#### Research Events and Incidents

In this trial, adverse events have been defined as any untoward medical occurrence in a trial participant, which does not necessarily have a causal relationship with the intervention. Serious adverse events are defined as any adverse event that results in death, is life-threatening (an event in which the participant was at risk of death at the time of the event; it does not refer to an event that hypothetically might have caused death if it were more severe), requires hospitalization (inpatient admission), or results in persistent or significant disability or incapacity.

The number, nature, and severity of serious adverse events (if any) will be reported separately by study arm at each follow-up time point. The number of participants who experience adverse events will likewise be reported separately by study arm.

## Results

This study was supported by a joint National Institute for Health Research UCLH Biomedical Research Centre grant (BRC789/OB/BD/110370) and Rosetrees Trust grant (UCL-Obesity-2020\101), funded in September 2020. Following initial protocol development, ethics approval, and trial setup, the clinical phase formally commenced with registration on June 16, 2023. Enrollment is ongoing, with a projected end date of March 2026. As of May 2025, a total number of 220 patients have been screened for participation, of whom 30 have enrolled in the trial. The first results are expected to be submitted for publication by mid-2026.

## Discussion

### Overview

This study is designed to evaluate the short-term physiological impact and acceptability of a telehealth-delivered prehabilitation program for patients preparing for MBS. We anticipate that the intervention will result in improvements in CRF over a 4-week period and will be acceptable to participants. These findings will inform refinements to the program and guide future implementation efforts.

The recruitment process for this study has been shaped by various logistical and contextual factors, including COVID-19–related surgical delays and considerations around participant engagement in exercise interventions. These factors highlight the need for flexible and accessible prehabilitation strategies for patients awaiting MBS. The use of telehealth for exercise delivery presents potential advantages in terms of accessibility and scalability, aligning with broader efforts to integrate remote interventions into routine clinical care. Although obesity-specific evidence on telehealth prehabilitation remains limited [55], insights from other clinical populations—such as those undergoing cancer treatment or orthopedic surgery—suggest that early investment in remote intervention models can yield long-term benefits in terms of program scalability and patient outcomes [56].

### Strengths and Limitations

A key strength of this study is its pragmatic design, which reflects real-world clinical pathways and enhances accessibility using telehealth. The use of CPET for our primary outcome provides a gold-standard, objective measure of physiological change. However, several limitations must be acknowledged. The absence of postoperative or long-term follow-up limits our ability to assess sustained changes in outcomes. While the use of usual care as a comparator reflects current clinical practice, its variability across settings can introduce confounding and may not adequately control for study-induced behavioral changes.

Findings from this study will inform refinements to the BARI-Prehab program, helping to optimize its implementation and sustainability. Participant feedback will be used to identify which components of the program are most valued, which may require adjustment, and how delivery can be improved. Future phases of the research will examine the acceptability of telehealth-based prehabilitation in greater depth and evaluate its integration within standard MBS pathways. While this study focuses on short-term outcomes, future studies will be designed to include postsurgical and longer-term follow-up to assess whether early improvements translate into enhanced perioperative recovery and sustained cardiometabolic benefits. Ultimately, this study may contribute to the development of scalable, evidence-based, preoperative exercise interventions that better support patients preparing for MBS.

### Dissemination Plan

Results from this study will be disseminated through peer-reviewed publications, conference presentations, and among clinical bariatric surgery providers. These efforts aim to support the translation of research into patient-centered prehabilitation practice.

## Appendix

Multimedia Appendix 1 Summary of Participant Communications and Behaviour Change Techniques.

Multimedia Appendix 2 Measured variables and timeline of outcomes.

## Abbreviations

AT: anaerobic threshold
CPET: cardiopulmonary exercise testing
CRF: cardiorespiratory fitness
MBS: metabolic and bariatric surgery
NHS: National Health Service
PA: physical activity
SPIRIT: Standard Protocol Items: Recommendations for Interventional Trials
UCL: University College London
UCLH: University College London Hospitals
