# Supplementary material for: Evaluating the Impact of Telehealth Exercise Prehabilitation on Cardiometabolic Health in Bariatric Surgery Candidates: Protocol for the BARI-Prehab Randomized Controlled Trial
Source: JMIR Res Protoc. 2025 Nov 13;14:e77538. doi: 10.2196/77538 (PMC12661224; doi:10.2196/77538)
Supplement: Multimedia Appendix 1 [file resprot_v14i1e77538_app1.docx]

**Table S2.** Summary of participant communications and behaviour change techniques.

| **Communication** | **Timing** | **Content Summary** | **Behaviour Change Techniques** |
| --- | --- | --- | --- |
| **Reminder email or SMS** | 24-48 hours before session | Session details and reminder, encouragement, goal reinforcement, contact info | Self-monitoring, social support, positive reinforcement |
| **Pre-Session Discussion** | Pre- supervised exercise | Health check-in, setting or review of goals, barrier identification | Goal setting, problem solving, self-monitoring |
| **Post-Session Discussion** | Post-supervised exercise | Acknowledgement of effort, personalised feedback, reflection, next goal planning | Feedback on performance, social reward, goal setting |
| **Confidence & Importance Rulers** | Every second session | Participants rate confidence and importance of physical activity (-5 to +5 scale) | Self-evaluation, motivational interviewing technique, enhancing autonomy and relevance |

This table aligns with the Behaviour Change Technique Taxonomy v1 [50] and integrates evidence-based strategies [48,49] shown to improve physical activity and engagement in populations with overweight and obesity.
