# Supplementary material for: Evaluating the Impact of Telehealth Exercise Prehabilitation on Cardiometabolic Health in Bariatric Surgery Candidates: Protocol for the BARI-Prehab Randomized Controlled Trial
Source: JMIR Res Protoc. 2025 Nov 13;14:e77538. doi: 10.2196/77538 (PMC12661224; doi:10.2196/77538)
Supplement: Multimedia Appendix 2 [file resprot_v14i1e77538_app2.docx]

**Table S1.** Measured variables and timeline of outcomes. This is a Multimedia Appendix to a full manuscript published in the J Med Internet Res. For full copyright and citation information see http://dx.doi.org/10.2196/jmir.77538.

| **Outcome** | **Instrument** | **Procedure and Data Processing** | **Baseline** | **Intervention** | **Post-**  **intervention** |
| --- | --- | --- | --- | --- | --- |
|  |  |  | **Visit #1** |  | **Visit #2** |
| Cardiorespiratory fitness  (VO_2AT_ and VO_2peak_ in ml.kg-1.min-1) | Cardiopulmonary Exercise Test (CPET): Metalyzer® 3B-R3; CORTEX Biophysik GmbH, Leipzig, Germany. This has been shown to have intraclass reliability coefficient for repeat measures of 0.984 for VO_2_, and 0.977 for VCO_2_ ^51^  Software: MetaSoft version 5.9.2 (1.2.6) (x64).  Electromagnetically braked cycle ergometer: Lode Corival 906900; Groningen, The Netherlands | CPET will involve non-invasive measurement of the cardiovascular and respiratory systems whereby participants exercise on a stationary bike until volitional fatigue. The test protocol involves a 3 minute rest period, followed by 3 minutes of unloaded exercise, then 8-12 minutes of appropriately ramped exercise (dependent on patient activity levels). Symptom-limited test-termination is desirable, but standard test-termination criteria^52^ will be adhered to. The facemask will enable continuous measurement of respiratory gas exchange.  Anaerobic threshold (AT) will be identified using the V-slope method.^44^ All data will be averaged (over 30 second intervals), smoothed (middle 5 of 7 breaths) and checked for calibration. | X |  | X |
| Resting metabolic rate (kcal/d) | Indirect calorimetry: Metalyzer® 3B-R3; CORTEX Biophysik GmbH, Leipzig, Germany | Participants will lie supine wearing a facemask harnessed over the mouth and nose. Gas exchange data will be recorded for 20 minutes, with the most stable 5 minute period (lowest coefficient of variation for VO₂ and VCO₂) selected for analysis, following best-practice guidelines.^53^ Resting metabolic rate (RMR) will be calculated using the Weir equation, with predictive equations^54,55^ used for comparison. | X |  | X |
| Muscle strength (kg) | Dynamometer: Grip-D, TKK-5401; Takei Scientific Instruments Co. Ltd., Niigata, Japan. This device provides reproducible outcomes with a mean difference of less than 1.0 kg for repeated isometric grip strength measures.^56^ | Grip strength will be assessed using a hand-held dynamometer. Participants will be instructed to hold the dynamometer in one hand, with the arm by the side of the body, and elbow bent at 90°. The handle of the dynamometer can be adjusted to allow for hand-size variation. The participant will be asked to squeeze the dynamometer with maximum force and maintain this for 3-5 seconds. The manoeuvre is repeated on the contralateral hand, with the highest value of three trials recorded. | X |  | X |
| Body mass (kg) | LOG507 Professional Medical (Digital) Scale | Body mass will be recorded with minimal clothing to the nearest 100 g on calibrated digital scales | X |  | X |
| Body composition (% body fat and FFM) | Bioelectrical Impedance Analysis (BIA) InBody body composition analyser (BWA 2.0 ® , InBody Corp., Seoul, Republic of Korea) | Body composition (%BF and FFM) will be assessed using InBody_770_ BIA which has a reported reliability of >0.98 for %BF and >0.99 for FFM^57^. | X |  | X |
| Height (cm) | Wall-mounted stadiometer, Seca 216 (Seca GmbH & Co. Hamburg, Deutschland) | Height will be assessed to the nearest 0.1 cm using international standards.^58^ | X |  |  |
| Resting heart rate (bpm) | 12-lead Electrocardiography (ECG) Custo diagnostic 4.5.0 Custo Med GmbH, Ottobrunn, Germany | 12-lead ECG will be fitted prior to indirect calorimetry and CPET. This will be used to assess resting heart rate and HRV, and to monitor safety during exercise testing. Raw ECG data will be exported and analysed for time- and frequency-domain HRV parameters following standardised guidelines. Artifacts will be manually checked and corrected where needed prior to HRV metric calculations. | X |  | X |
| Heart-rate variability (ms) |  |  | X |  | X |
| Physical activity (7-day PA and sedentary behaviour) | Accelerometry (Actigraph wGT3X-BT and ActiLife software (ActiGraph LLC, Pensacola FL). This instrument has been used extensively in clinical research due to its accuracy, reliability, and practicality.^59^ | Accelerometer data will be downloaded and processed using *ActiLife* software (ActiGraph LLC, Pensacola, FL). Data from the hip-worn ActiGraph wGT3X-BT devices will be sampled at 30 Hz and integrated into 60-second epochs. Non-wear time will be defined as 60 minutes of consecutive zero counts, allowing for up to 2 minutes of counts between 0 and 100. A valid day will consist of ≥10 hours of wear time, and participants must have at least 4 valid days for inclusion in the analysis. Wear time is determined by subtracting non-wear time from 24 hours. PA will be categorised into sedentary, light, moderate, and vigorous intensity using established cut points. |  | X |  |
| Adherence | Record in case-report file (investigator) | Attendance at pre- and post-intervention testing sessions  Adherence will be calculated as the proportion of starting participants who also attend the 4-week follow-up assessment. | X |  | X |
| Compliance (# sessions completed / 7 and # self-led sessions completed) | (1) Record in case-report file (investigator), and (2) exercise diary (self-report) | Compliance will be quantified using two categories: (1) attendance in the online supervised PA sessions, and (2) achievement of 30 min of MVPA/day on days where exercise has been prescribed. Data will be reported as the proportion of ‘prescribed PA days’ where participants were compliant within each of the categories across the four-week period. |  | X |  |
| Acceptability | ‘Participant Satisfaction’ survey | Likert scale questions and open-ended responses. |  |  | X |
